# Utility of Clinical–Radiomic Model to Identify Clinically Significant Prostate Cancer in Biparametric MRI PI-RADS V2.1 Category 3 Lesions

**DOI:** 10.3389/fonc.2022.840786

**Published:** 2022-02-24

**Authors:** Pengfei Jin, Liqin Yang, Xiaomeng Qiao, Chunhong Hu, Chenhan Hu, Ximing Wang, Jie Bao

**Affiliations:** ^1^ Department of Radiology, The First Affifiliated Hospital of Soochow University, Suzhou, China; ^2^ Institute of Medical Imaging, Soochow University, Suzhou, China

**Keywords:** radiomics, clinically significant prostate cancer, PI-RADS score 3, nomogram, biparametric MRI (Bp-MRI)

## Abstract

**Purpose:**

To determine the predictive performance of the integrated model based on clinical factors and radiomic features for the accurate identification of clinically significant prostate cancer (csPCa) among Prostate Imaging Reporting and Data System (PI-RADS) 3 lesions.

**Materials and Methods:**

A retrospective study of 103 patients with PI-RADS 3 lesions who underwent pre-operative 3.0-T MRI was performed. Patients were randomly divided into the training set and the testing set at a ratio of 7:3. Radiomic features were extracted from axial T2WI, diffusion-weighted imaging (DWI), and apparent diffusion coefficient (ADC) images of each patient. The minimum redundancy maximum relevance (mRMR) and least absolute shrinkage and selection operator (LASSO) feature selection methods were used to identify the radiomic features and construct a radiomic model for csPCa identification. Moreover, multivariable logistic regression analysis was used to integrate the clinical factors with radiomic feature model to further improve the accuracy of csPCa identification, and the two are presented in the form of normogram. The performance of the integrated model was compared with radiomic model and clinical model on testing set.

**Results:**

A total of four radiomic features were selected and used for radiomic model construction producing a radiomic score (Radscore). Radscore was significantly different between the csPCa and the non-csPCa patients (training set: *p* < 0.001; testing set: *p* = 0.035). Multivariable logistic regression analysis showed that age and PSA could be used as independent predictors for csPCa identification. The clinical–radiomic model produced the receiver operating characteristic (ROC) curve (AUC) in the testing set was 0.88 (95%CI, 0.75–1.00), which was similar to clinical model (AUC = 0.85; 95%CI, 0.52–0.90) (*p* = 0.048) and higher than the radiomic model (AUC = 0.71; 95%CI, 0.68–1.00) (*p* < 0.001). The decision curve analysis implies that the clinical–radiomic model could be beneficial in identifying csPCa among PI-RADS 3 lesions.

**Conclusion:**

The clinical–radiomic model could effectively identify csPCa among biparametric PI-RADS 3 lesions and thus could help avoid unnecessary biopsy and improve the life quality of patients.

## Introduction

Prostate cancer (PCa) is one of the most common cancer and the second leading cause of cancer deaths among men (1). The Prostate Imaging Reporting and Data System (PI-RADS) aims to standardize the interpretation and reporting of prostate MRI, which develop a 5-point assessment to assist in identifying suspicious lesions and reflect their relative possibility of a clinically significant prostatic cancer (csPCa) (2). Despite offering valuable information in predicting csPCa on a population level, one of the main limitations of PI-RADS is the high inter-reader variability impacting on cancer detection (3). Additionally, the PI-RADS has an inability to resolve some ambiguity and uncertainty associated with some reporting criteria and lesion descriptors.

Currently, the classification of PI-RADS category 3 lesions has not been clearly defined, which represents a “gray zone” that contains benign, indolent, and invasive lesions. Since considered as positive MRI finding, PI-RADS 3 lesion should always be biopsied according to European Association of Urology guidelines, which results in a diagnosis of csPCa in 3%–50% of the patients (4, 5). However, other studies have reported that cancer diagnosis rates range from 2% to 23% in PI-RADS 3 lesions and suggested that mostly they are benign lesions or non-significative cancers (6–8). Due to the uncertainty and lack of clear management recommendations of undetermined lesions, it is still under debate whether a biopsy should be performed or not. Therefore, determining which lesions are csPCa will help improve patients’ quality of life *via* avoiding unnecessary biopsies and overtreatment.

Recent studies have shown that patient age, high prostate-specific antigen density (PSAD), PSA velocity, low apparent diffusion coefficient (ADC) signal, and even genetic risk are associated with the existence of csPCa (5, 7, 9). Furthermore, it has been illustrated that in radiomics, a large number of quantitative features, extracted from MRI images, have been employed in detecting PCa, evaluating PCa aggressiveness, and clinical decision-making (10). However, the potential role of MRI radiomics in identifying csPCa among PI-RADS 3 lesions has not been determined. A recent study suggested that texture analysis based on machine learning could help to identify csPCa in PI-RADS 3 lesions (11). In 2019, PI-RADS V2.1 proposed the concept of biparametric magnetic resonance imaging (bpMRI), which only includes T2WI, diffusion-weighted imaging (DWI), and ADC sequences to simplify the process of prostate MRI scanning (2). The diagnostic accuracy and performance of bpMRI are comparable to multi-parameter magnetic resonance imaging (mpMRI), which also covers dynamic contrast enhancement (DCE), and the former is less expensive, rapid, and well tolerated by patients (12).

Therefore, the objective of this study was to construct a nomogram that integrate radiomics based on bpMRI and clinical information to identify csPCa in PI-RADS 3 lesions.

## Materials and Methods

### Study Cohort

The Institutional Review Board approved this retrospective study performed at a single medical institution and waived the requirement of informed consent (2021; Approval No. 262). A retrospective collection of 1,675 patients who underwent prostate MRI examination due to PSA elevation in our hospital from January 2016 to January 2019 was conducted. All MRI images were assigned PI-RADS V2.1 score by two genitourinary radiologists experienced in urological diagnosis (3- and 6-year experience). Screened out lesions identified as PI-RADS 3 and sent to another experienced genitourinary radiologist (more than 10-year experience) for review. All the radiologists were blind to the histopathological results at the time of reading. If there is a disagreement on the diagnosis, the three radiologists will discuss it until a consensus is reached. PI-RADS V2.1 defined the score 3 lesion as the focal low signal of ADC and/or high signal of DWI with high b-value in the peripheral zone (PZ) or the uneven signal of T2WI sequence in the transitional zone (TZ) with blurred edges. In the course of the discussion, 25 cases were downgraded to PI-RADS 1–2, and 39 cases were upgraded to PI-RADS 4–5. The final scoring results showed that 859 patients (51.3%) had PI-RADS scores of 1 and 2, and 153 patients (9.1%) had PI-RADS scores of 3; the other 663 cases (39.6%) had PI-RADS scores of 4 and 5.

**Figure 1:**
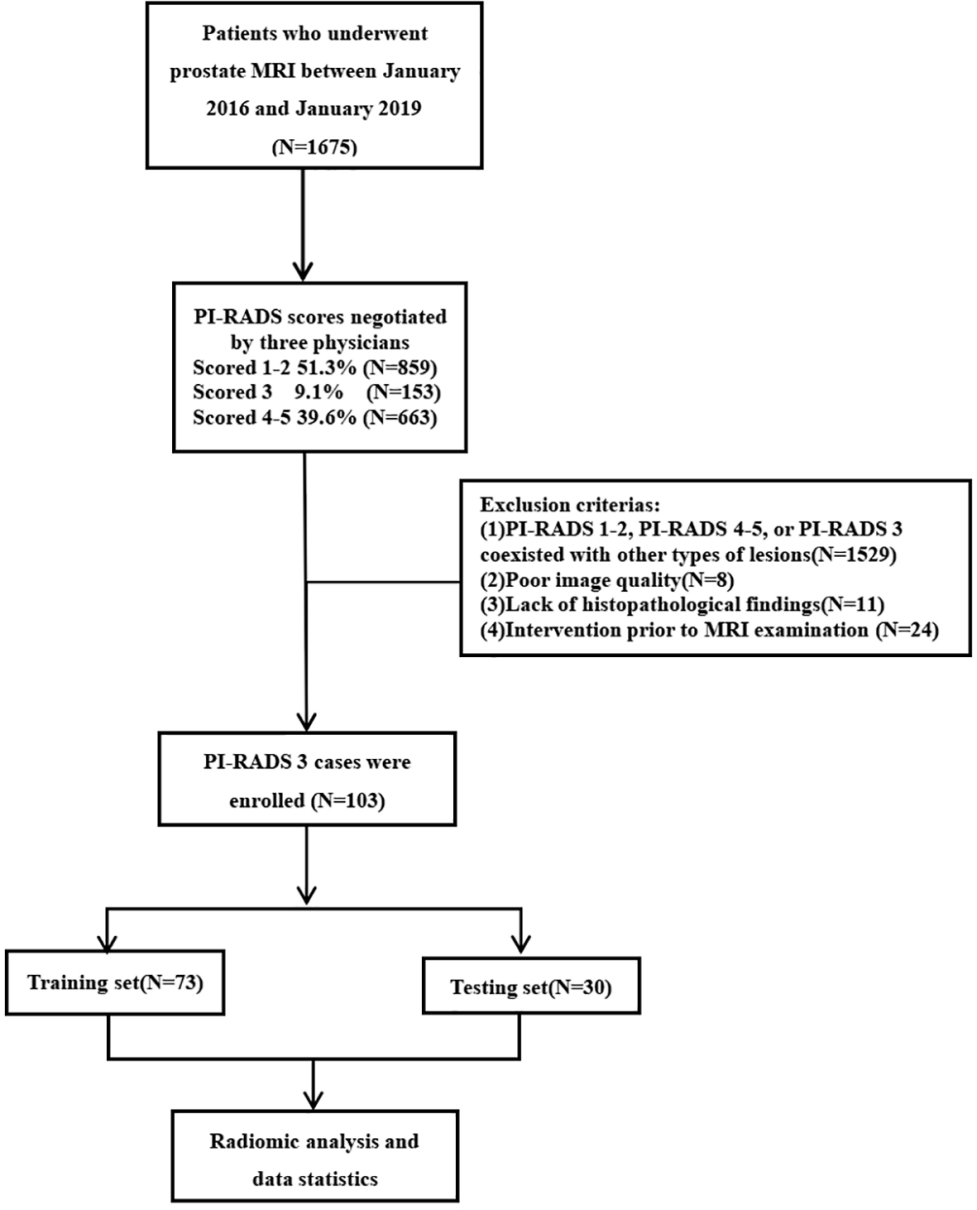
Patient recruitment flowchart.

Exclusion criteria were as follows: (1) PI-RADS 1–2, PI-RADS 4–5, or PI-RADS 3 coexisted with other types of lesions (N = 1,529); (2) poor image quality (N = 8); (3) MRI findings were not confirmed by histopathological results(N = 11); (4) intervention prior to MRI examination, such as biopsy, surgery, or hormone therapy (N = 24) (**Figure 1**). In addition, clinical data, such as the International Society of Urological Pathology (ISUP) grade, pathological stage, age, and PSA value, were obtained by querying electronic records in PACS system. A total of 103 patients meeting above criteria were included in this study, with an average age of 67.5 ± 9.4 years and a mean PSA level of 14.9 ± 13.8 ng/ml.

### Scanning Equipment and Parameters

A 3.0-T superconducting MR scanner (Skyra; Siemens, Munich, Germany) with 32-channel body phased line coil was adopted. The scanning sequence included T1WI, axial T2WI (no fat-saturated), sagittal T2WI, coronal T2WI, DWI (b = 100, 800, 1 500 s/mm^2^) and/or dynamic-contrast-enhanced T1WI. Based on the DWI images of 1,500 b-values, the ADC icons were calculated by extended single exponential fitting model. The specific scanning parameters were recommended by PI-RADS V2.1 (**Table 1**) (2).

**Table 1:** Multi-parameter MRI scan sequence and parameters.

Sequence	Repetition time (ms)	Echo time (ms)	Layer thickness (mm)	Interlayer spacing (mm)	Field of view (mm × mm)
T1WI	680.0	13.00	5	0.50	380 × 380
Axial T2WI	6,980.0	104.00	3	0	200 × 200
Sagittal T2WI	3,900.0	89.00	3	0.45	200×200
Coronal T2WI	3,500.0	85.00	3	0.60	220 × 220
DWI	5,000.0	72.00	3	0	288 × 288
DCE-MRI	4.2	1.34	3	0	260 × 260

T2WI, T2-weighted imaging; DWI, diffusion-weighted imaging; ADC, apparent diffusion coefficient; DCE, dynamic contrast enhancement.

### Biopsy and Histopathology

All identified patients underwent MRI-transrectal ultrasound (MRI-TRUS) fusion-guided prostate biopsy with a navigation system (HIVISION Noblus/TopicPath). At least two biopsy samples were obtained from each targeted lesion. The 12-core systematic biopsies were routinely performed following the targeted biopsy procedure. If patients underwent radical prostatectomy, their pathological ISUP grades, instead of biopsy ISUP grades, were used to define csPCa. The pathological results were evaluated by expert urologists, and the location of the lesions were recorded to ensure the correspondence to the suspicious lesions on MRI. csPCa was defined as ISUP Class 2 or higher (Gleason = 3 + 4 or higher). Lesions with GS = 3 + 3 were defined as clinically insignificant prostate cancer (ciPCa), which were categorized in the same group as benign lesions (13).

### MRI Image Preprocessing and Focus Segmentation

First, histogram-based intensity standardization method was used to standardize the bpMRI, and the voxel size of image was resampled to 1 mm × 1 mm × 1 mm. Then, the axial T2WI, high b-value (1,500 mm/s^2^) DWI, and ADC were co-registered using Elastix software package (v.4.10) to ensure that DWI and ADC images have the same resolution, field of view (FOV), and orientation as T2WI. The two radiologists involved in the image evaluation used Insight Segmentation and Registration Toolkit (ITK, v. 4.7.2; https://itk.org/) to manually draw the region of interest (ROI) on the T2WI image layer by layer, then copy it to DWI and ADC images to ensure the consistency of volume of interest (VOI) sketches in different sequences. To ensure the stability and repeatability of the annotations, the same radiologists repeated the annotation procedure after a week, and all the annotations were re-examined by another senior radiologist with 15 years of experience in prostate MRI diagnosis.

### Feature Extraction and Consistency Agreement

The radiomics software FeAture Explorer (FAE v0.4.0) was used to extract and select the features of each mode with reference to VOI (14). A total of 2,553 radiomic features were extracted, including (1) 54 first-order gray statistics, (2) 42 features of shape-based, (3) 72 gray-level co-occurrence matrixes (GLCM), (4) 48 gray-level run length matrixes (GLRLM), (5) 48 gray-level size zone matrixes (GLSZM), (6) 42 gray-level dependence matrixes (GLDM), and (7) 15 neighborhood gray tone difference matrixes (NGTDM). In addition, 2,232 wavelet features were extracted in three spatial directions. The inter- and intra-observer reproducibility of tumor segmentation and feature extraction was evaluated by intraclass correlation coefficients (ICCs), and radiomic features with ICCs values >0.75 were retained.

### Radiomics Model Development

All samples were randomly divided into the training set and the testing set at a ratio of 7:3. First of all, most of the features were excluded by using the mRMR method, and only 30 features with the least redundancy and the greatest correlation with the target label were retained. The radiomic signature was constructed based on the features selected from the training set. Due to the large number of features extracted, there was redundancy between features, and some features had little or no correlation with the modeling object. Then, least absolute shrinkage and selection operator (LASSO) was used to select the most useful features. Finally, a logistic regression was trained using the remaining features, and a radiomic score was calculated as the linear combination of the selected radiomic features and corresponding coefficients.

In addition, we have employed the multivariate logistic regression analysis to combine clinical characteristics, including age, PSA value, zone of the lesion, prostate volume, with radiomic features, and identify the independent predictors of csPCa among PI-RADS V2.1 category 3 lesions in the training set. Furthermore, a clinical–radiomic nomogram was built to provide clinicians with a quantitative tool for csPCa identification. The area under receiver operating characteristic (ROC) curve (AUC) was adopted to evaluate the accuracy of the nomogram in identifying csPCa.

### Model Evaluation

AUCs of clinical, radiomic, and clinical–radiomic model were basically evaluated with histopathological manifestations in both training and testing set. Moreover, DeLong’s test was used to compare the ROC curves of the nomogram, radiomic, and clinical model, and Hosmer–Lemeshow test was performed to evaluate the goodness-of-fit of the calibration curve between nomogram and the pathological results. Furthermore, decision curve analysis (DCA) was used to quantify the net benefit of each predictive model.

### Statistical Analysis

To identify csPCa among PI-RADS 3 lesions on bpMRI, we trained the binary classification models by labeling csPCa as 1 and both ciPCa and benign lesions as 0. R language software (version 4.1.0, www.Rproject.org) was used for quantitative feature analysis. The Shapiro–Wilk test was performed to evaluate the normality of data. According to the results of normality test, independent sample t-test or Mann–Whitney U-test was used to detect the difference of clinical characteristics between non-csPCa group and csPCa group. Multivariable logistic regression was performed using “rms” package to construct the clinical–radiomic nomogram. ROC curves and AUCs were established using “PROC” package to evaluate the diagnostic accuracy of each predictive model. Calibration curve and Hosmer–Lemeshow test were performed with “ModelGood” and “DescTools” packages, respectively. Finally, the “rmda” software package was used for decision curve analysis. *p* < 0.05 indicated that the difference was statistically significant.

## Results

### Baseline Characteristics

Among the 103 lesions with a score of PI-RADS 3 were 28 cases of csPCa (27.2%), 70 cases of benign hyperplasia (67.9%), and 5 cases of ciPCa (4.9%); 44.7% (46/103) lesions were located in TZ, and 55.3% (57/103) lesions were located in PZ. The prevalence of csPCa located in TZ and PZ was 11.7% (12/103) and 15.5% (16/103), respectively. Among the 28 patients with csPCa, 13 cases were ISUP grade 2 (GS = 3 + 4), 6 cases were ISUP grade 3 (GS = 4 + 3), 7 cases were ISUP grade 4 (GS = 4 + 4, 5 + 3), and 2 cases were ISUP grade 5 (GS = 5 + 4). The diameter of lesions ranged from 5.2 to 18.6 mm, with an average diameter of 9.5 mm ± 3.4 mm (**Table 2**).

**Table 2:** Demographic and disease characteristics.

	Training set	Testing set	*p*-value
Ages	64.7 ± 9.2	66.8 ± 8.3	0.326
PSA (ng/ml)	14.8 ± 10.1	17.5 ± 5.7	0.518
Lesion type			–
Benign	48 (46.6%)	22 (21.4%)	
csPCa	22 (21.4%)	6 (5.8%)	
ciPCa	3 (2.9%)	2 (1.9%)	
Zone			
PZ	41	16	
TZ	32	14	
Total	73	30	

PSA, prostate-specific antigen; csPCa, clinically significant prostate cancer; ciPCa, clinically insignificant prostate cancer; PZ, peripheral zone; TZ, transitional zone.

The p-values are derived from the comparison between training set and testing set.

### Performance of Prediction Models

#### Clinical Model

Univariate logistic analysis showed that age and PSA value were significant factors for predicting csPCa. Multivariate logistic regression analysis showed that the odd ratio of PSA level to detect csPCa was 1.04; the difference was statistically significant (*p* = 0.041). Therefore, these two clinical factors can be used as independent predictors (**Table 3**). Finally, a logistic regression classifier was established according to the selected clinical features. The performance of clinical model in identifying csPCa is listed in **Table 4**.

**Table 3:** Univariate and multivariate logistic analyses results of clinical factors.

Baseline characteristics	Non-clinically significant cancer (n = 75)	Clinically significant cancer (n = 28)	Univariate logistic regression		Multivariate logistic regression	
			Odds ratio (95%CI)	*p*-value	Odds ratio(95%CI)	*p*-value
Age	65.6 ± 9.1	72.5 ± 8.3	1.06 (1.00–1.13)	0.042	1.09 (1.00–1.13)	0.046
PSA (ng/ml)	12.3 ± 10	21.8 ± 19.4	1.04 (1.00–1.09)	0.034	1.04 (1.00–1.08)	0.041
Lesion location			1.01 (1.00–1.07)	0.063	1.03 (1.02–1.10)	0.052
Peripheral zone	41 (39.8%)	16 (15.5%)				
Transition zone	34 (33.0%)	12 (11.7%)				
Gland volume	43.8 ± 24.3	40.7 ± 15.8	1.02 (0.97–1.06)	0.074	1.01 (0.99–1.05)	0.097

PSA, prostate-specific antigen; 95%CI, 95% confidence interval.

**Table 4:** The AUC outcomes of clinical, radiomic, and combined model in prediction of csPCa in category 3 lesions.

	Clinics		Radiomics		Nomogram	
Index	Training set	Testing set	Training set	Testing set	Training set	Testing set
Cutoff	−0.77		−0.84		−1.54	
Accuracy (95%CI)	0.74 (0.62–0.84)	0.57 (0.54–0.88)	0.75 (0.64–0.85)	0.57 (0.37–0.75)	0.78 (0.67–0.87)	0.70 (0.45–0.82)
Sensitivity	0.68	0.67	1.00	1.00	0.91	0.83
Specificity	0.76	0.75	0.65	0.46	0.73	0.65
PPV	0.56	0.40	0.55	1.00	0.59	0.47
NPV	0.85	0.90	0.46	0.32	0.95	0.91
AUC (95%CI)	0.70 (0.55–0.84)	0.85 (0.68–1.00)	0.85 (0.76–0.93)	0.71 (0.52–0.90)	0.90 (0.83–0.97)	0.88 (0.75–1.00)
*p*-value (vs nomogram)	*p* = 0.001	*p* = 0.048	*p* < 0.001	*p* < 0.001	–	–

95%CI, 95% confidence interval; PPV, positive predict value; NPV, negative predictive value. The p-values from Delong tests compared with nomogram.

#### Prediction Model Based on Radiomic Features

**Figure 2:**
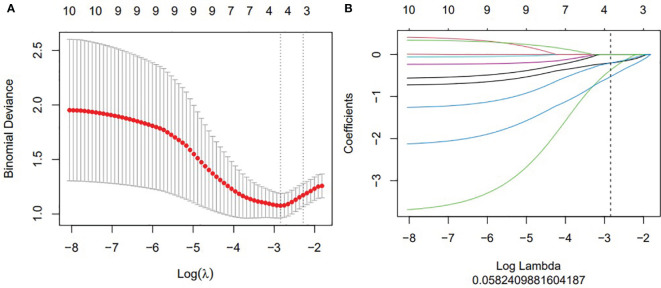
The construction of LASSO regression model. **(A)** Curve of binomial deviation of biparameter MR radiomics model varying with parameter λ. The horizontal axis is the log (λ) value. The vertical axis represents binomial deviation. The number above represents the number of selected features, and the λ at the minimum binomial deviation of the model is the optimal value (vertical dotted line). **(B)** Biparameter MRI model changing with λ. The number above indicates the number of features filtered out.

**Figure 3:**
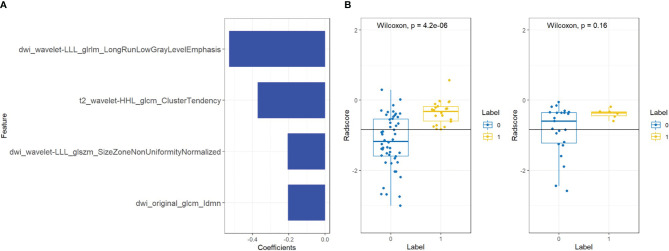
Features and radiomics labels used in bpMRI model. **(A)** Imaging characteristics screened by bpMRI model. **(B)** comparison of Radscore between training set (left) and testing set (right). The blue label represents benign lesions or ciPCa, and the yellow label is csPCa.

A total of four radiomic features were selected and used to build a logistic regression model based in the training cohort (**Figures 2** and **3**). Radscore was significantly different between the csPCa group and the non-csPCa group (training set: *p* < 0.001; testing set: *p* = 0.035), which indicated that the probability of csPCa was positively correlated with Radscore. The radiomics feature model has an above-average predictive efficiency for csPCa in PI-RADS 3 lesions, with an AUC of 0.71 (**Table 4**).

#### Clinical–Radiomic Model

**Figure 4:**
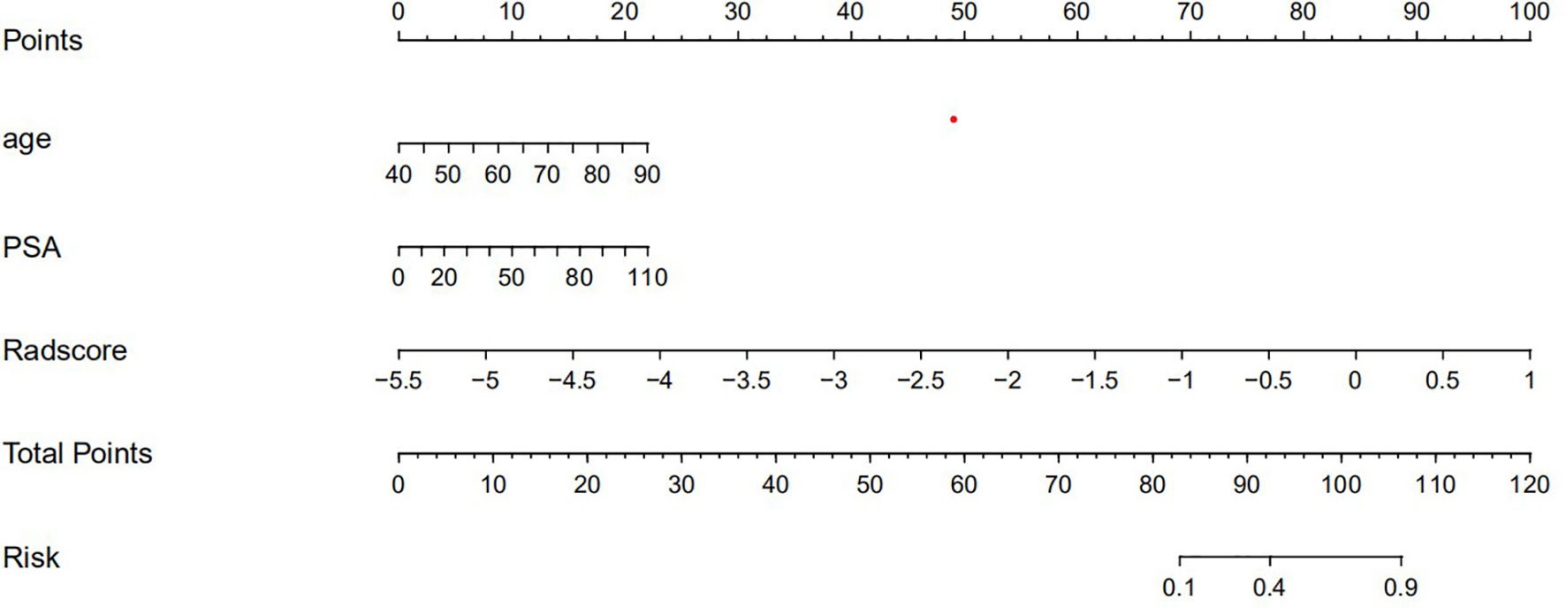
Clinical–radiomic nomogram.

**Figure 5:**
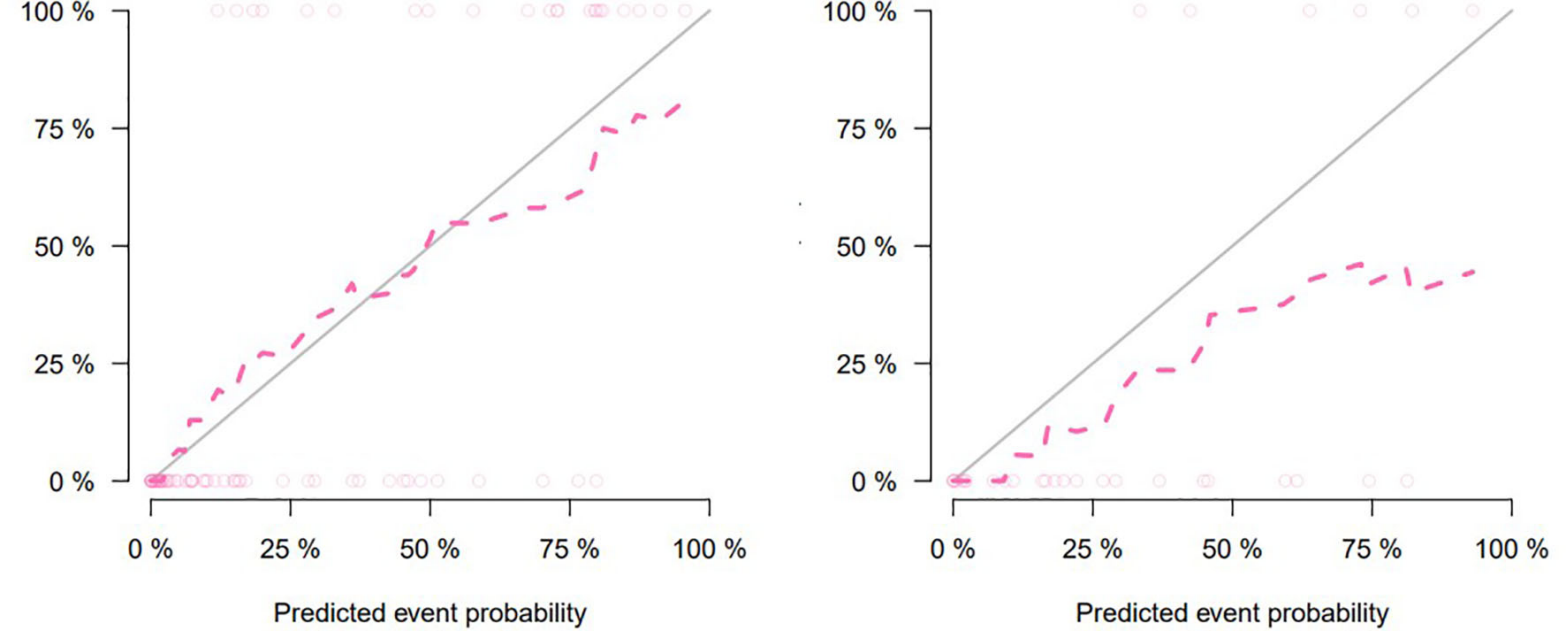
Calibration curve for clinical–radiomic nomogram prediction of the consistency between the predicted results and pathological results (training set on the left, testing set on the right).

**Figure 6:**
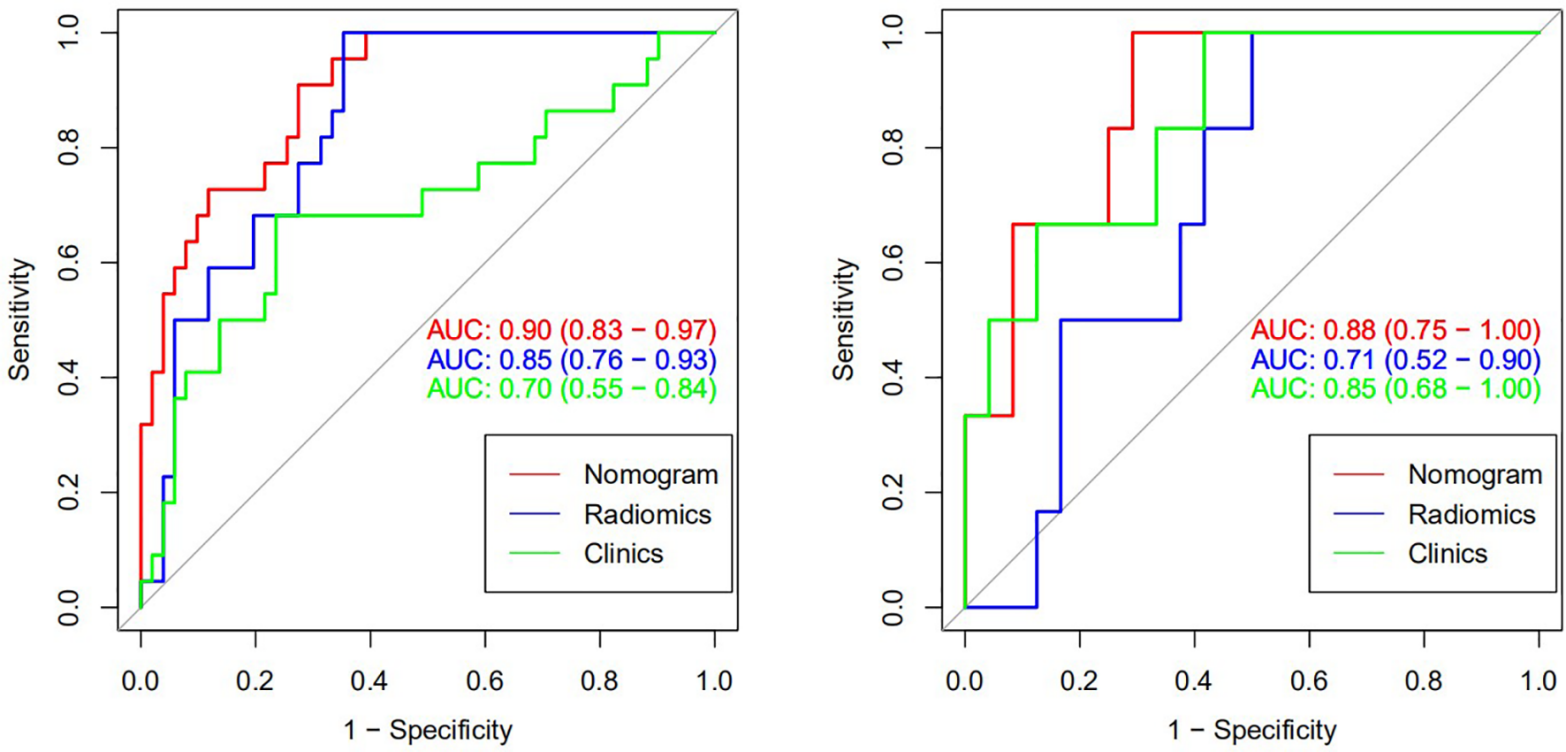
Receiver operating characteristic (ROC) curve of csPCa predicted by three models (training set on the left and verification set on the right).

**Figure 7:**
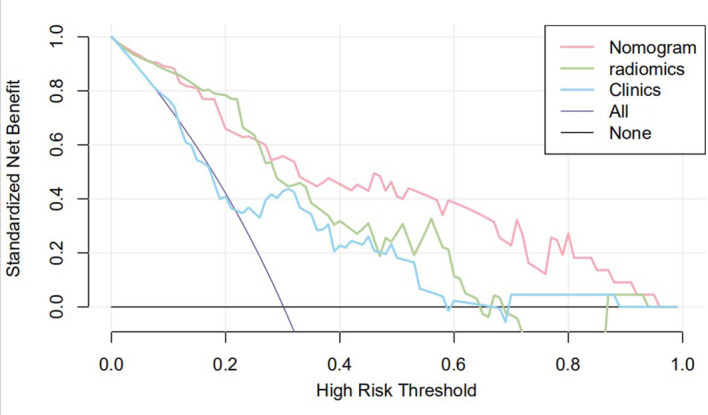
Clinical decision curve of the three models. The X-axis represents the threshold probability, and the Y-axis represents the net benefit. The decision curve showed that if the threshold probability of a patient was within the range from 25% to 95%, using the joint nomogram to predict csPCa occurrences added more benefit than the biopsy-all-patients scheme or the surveil-all-patients scheme.

The nomogram that combined age, PSA, and Radscore is shown in **Figure 4**. Compared with the radiomics model, the clinical–radiomic nomogram showed an improved performance in predicting csPCa among PI-RADS category 3 lesions. The AUC values of the training group and the validation group were 0.90 (95%CI: 0.83–0.97) and 0.88 (95%CI: 0.75–1.00), respectively. The calibration curve showed that the nomogram had a higher pathological coincidence rate. The p-value of the nomogram prediction ability obtained by the Hosmer–Lemeshow test was 0.740 in the training cohort and 0.503 in the testing cohort (**Figure 5**). The AUC, accuracy, sensitivity, and specificity of the three models are listed in **Table 4**. Our results showed that the ability of nomogram to distinguish csPCa from non-csPCa in PI-RADS category 3 lesions was higher than that of clinical model and radiomic model. Furthermore, nomogram had the highest net benefit compared to clinical and radiology (**Figures 6** and **7**).

## Discussion

Radiomics is a technique for extracting and analyzing quantitative features from medical images. It can capture sub-visual signatures, such as the change of gray level and spatial distribution of the intensity. It has been shown that radiomics was of great potentials in PCa classification, risk stratification, and thus help with the clinical diagnostic workflow. The correlation between multi-parameter MRI radiomic features and Gleason grading also showed that the radiomic model could predict Gleason score and distinguish between invasive PCa (GS ≥ 4 + 3) and inert PCa (GS < 4 + 3). Zhang et al. developed and verified a non-invasive radiomic model based on MRI to distinguish between inert and invasive PCa before treatment. They finally selected nine radiomic features to construct imaging tags with 0.944 for sensitivity, 0.786 for specificity, and 0.901 for AUC in the validation set (15). Radiomic not only can assist PCa detection and grading but also can be used to evaluate tumor extracapsular invasion, which is conducive to accurate preoperative staging. Ma et al. selected 17 imaging features extracted from T2WI images to predict extracapsular invasion in PCa patients, showing great recognition ability and excellent calibration performance in training and verification sets (16).

Giambeluca et al. introduced the concept of texture analysis into the study of PI-RADS 3 lesions for the first time (11). They found that nine and six independent texture features on T2WI and ADC maps were significantly correlated with the final histopathological results, and the derived model predicted that the AUC of csPCa was 0.82 and 0.74, respectively. However, the sample size of their study is too small (only 46 PI-RADS 3 lesions) and has not been verified by the test set, which affects the reliability of the results. In this study, we constructed and validated a comprehensive diagnostic model combining clinical variables and radiomic features, which was used to identify csPCa lesions in PI-RADS 3 lesions on biparameter MRI, and compared with separate clinical model and radiomic model (Radscore). As demonstrated by DeLong’s test, the clinical–radiomic model was significantly superior to both clinical and Radscore in identifying csPCa. In the testing set, compared with 33% (2/6) in the clinical model, only 17% (1/6) csPCa were missed using the combined model, which helped reducing the rate of missed diagnosis greatly without significantly decreasing the specificity (75% vs. 65%). It is worth noting that although the radiomic model has a high sensitivity (100%), its specificity is very low (46%), which may lead to unnecessary biopsies. The combined model has the advantages of both clinical and radiomic model, and the output results have higher stability. In order to more intuitively show the risk probability of the comprehensive model for predicting csPCa, this study presents it in the form of nomogram. This clinical–radiomic nomogram provides an easy-to-use, quantifiable, and individualized screening tool for PCa, helping to avoid unnecessary treatment and invasive examination in men with PCa patients and preventing and delaying the progression of low-grade PCa. In recent years, the nomogram prediction model has been widely used in clinical medicine, using risk scores to represent the risk factors of a variety of diseases and predict the prognosis of patients. The expression of this model is clear, concise, easy to understand, and conducive to doctor–patient communication.

Previous studies on intelligent diagnosis of PI-RADS 3 lesions were limited to simple imaging features (17–19), without considering the additional diagnostic value of clinical indicators. Compared with these similar studies, this study fused clinical indicators and imaging features when designing the model and proved that the two are complementary in the differentiation of benign and malignant prostate lesions. Univariate and multivariate analyses showed that radiomic features, age, and PSA could be used as independent predictors for the differential diagnosis of benign and malignant prostate lesions. In this study, the AUC (0.85) of the radiomics model in the training set was higher than that of the clinical model (0.70), but in the testing set, the AUC of the radiomic model in the diagnosis of csPCa was lower than that of the clinical model (0.71 vs. 0.85, *p* < 0.05). In our opinion, the reason may be that the sample size of csPCa in the testing set was too small (only six cases) to accept comprehensive verification, thus caused a certain randomness in the result. In addition, malignant epithelial cells of sectional csPCa in PI-RADS 3 lesions were sparsely arranged and distributed along the acinar, which overlapped greatly with some benign diseases such as inflammation, hyperplasia, and fibrosis, resulting in insignificant changes in MRI signal. Age was associated with Gleason score, and the older the age, the higher the risk of poor histology. Studies have shown that the ORs and 95%CI of poor histological prognosis of prostate were 2.21 (1.30–3.76) and 1.58 (0.90–2.76) in men over 80 years old compared with those under 70 years old, respectively (20). Although this study did not prove a positive correlation between prostate volume and the occurrence of csPCa, several studies have confirmed that PSAD (PSA value/gland volume) was independently correlated to csPCa even in patients with serum PSA slightly above limits or even within normal limits, which was observed in every clinical scenario early diagnosis, repeat biopsy, and active surveillance (21). For example, Roscigno et al. found that higher PSAD was associated with higher risk of reclassification at confirmatory or follow-up biopsy using 0.20 as cutoff (22). Pagniez et al. increased the negative predictive value of PI-RADS from 84.4% to 90.4% by using the PSAD with a cutoff of 0.15 ng/ml/cc (23). PSAD is also useful to identify patients with elevated PSA due to PCa rather than intraprostatic inflammation, which is indeed a strong predictor of the absence of PCa in biopsy specimen (24). As a rule, compared with TZ, the risk of developing PCa tends to appear on PZ; therefore, the anatomical location of lesions is helpful for the differential diagnosis of equivocal lesions (defined as PI-RADS 3). Yang et al. analyzed cancer detection rate in 683 patients with PI-RADS 3 lesions of the PZ and TZ and reported 18.7% of csPCa in the PZ, while in the TZ, the rate of csPCa was 6.0% (25). However, the results of this study suggest that the zone of the lesions cannot be an independent predictor to refine classification of PI-RADS 3 lesions. The reason may be that the T2WI scoring criteria of PI-RADS 3 lesion in TZ is too vague to grasp for inexperienced evaluators; more TZ lesions were selected while building the study cohort, and the probability of csPCa increased accordingly. In addition, the clinical variables used to construct nomogram were not comprehensive enough, such as palpable nodule, correlation between PSA, and prostate volume, which were not analyzed. Studies have shown that despite low PSA levels, the incidence of csPCa is higher in patients with positive digital rectal examination (DRE) (26). PSA density and DRE could be analyzed in the nomogram as the next step to improve selection of PI-RADS 3 lesions. The decision curve also indicates that if the patient’s threshold probability is 25%–95%, patients can benefit more from using the radiomic feature-based nomogram in this study to predict the identification of benign and malignant nodules, and the combined model has better predictive performance than individual clinical risk factors or radiomics features.

In the construction of radiomics model, the feature subsets extracted from DWI images were the most relevant factor for classification of PI-RADS category 3 lesions, while the contribution of ADC feature subsets was the least, which was different from the previous research results of Bonekamp et al. They believe that the effectiveness of radiomic model based on bpMRI in distinguishing benign and malignant prostate lesions is comparable to that of single ADC model (27). The reason for the inconsistent results may be that the PI-RADS category 3 refers to the lesions with mild or moderate low signal on the ADC maps, excluding those obvious benign and malignant lesions in advance. In addition, the transitional zone lesions included in this study accounted for 44.7%, while the TZ lesions were mainly based on the definition of T2WI manifestations, and ADC had little reference significance in the diagnosis of TZ lesions.

For the generalization ability of the model across different populations, studies have shown that the performance of MRI to predict the presence of extraprostatic extension and high-grade PCa is unaffected in Caucasian and African American men, and no difference was found between races in pathological outcomes after radical prostatectomy (28). These findings suggest that access to and use of advanced diagnostic tests may help mitigate PCa racial disparities; thus, the present model may be valid also in other populations. In subsequent studies, independent external validation sets can be set up to evaluate the stability of the predicted results of the model in different cohorts.

There are some limitations in this study. First of all, this study was a retrospective analysis of a relatively small group of patients from a unitary institution and a single scanner, and our predictive model needs to be prospectively validated in a larger scale of patients from other medical units using different MRI scanners prior to wider clinical application. Second, clinical factors only analyzed PSA and age; rather, they may be used to discover a population at higher risk to have a csPCa. Therefore, increase probability of favorable results independently of imaging interpretation. Third, subjects were not followed up, so the number of cancers diagnosed in the months after baseline is not available. Finally, in this study, there was no separate analysis of PZ and TZ lesions.

## Conclusion

We have developed a nomogram based on radiomics and clinical indicators, which has excellent predictive performance for csPCa in biparametric PI-RADS 3 lesions, and provide an intuitive and quantitative method for radiologists to diagnose PCa more confidently and reduce unnecessary biopsies.

## Data Availability Statement

The raw data supporting the conclusions of this article will be made available by the authors, without undue reservation.

## Ethics Statement

The studies involving human participants were reviewed and approved by Institutional Review Board of the First Affiliated Hospital of Soochow University. Written informed consent for participation was not required for this study in accordance with the national legislation and the institutional requirements.

## Author Contributions

XW and JB created the study design. PJ collected the data and processed the data. PJ and LY conducted data analysis. PJ and LY wrote the manuscript. All authors contributed to the article and approved the submitted version.

## Funding

This study was supported by (1) Special Program for Diagnosis and Treatment Technology of Clinical Key Diseases in Suzhou (LCZX202001), (2) Gusu Health Talent Project of Suzhou (GSWS2020003), (3) Suzhou Key Laboratory of Health Information Technology (SZS201818).

## Conflict of Interest

The authors declare that the research was conducted in the absence of any commercial or financial relationships that could be construed as a potential conflict of interest.

## Publisher’s Note

All claims expressed in this article are solely those of the authors and do not necessarily represent those of their affiliated organizations, or those of the publisher, the editors and the reviewers. Any product that may be evaluated in this article, or claim that may be made by its manufacturer, is not guaranteed or endorsed by the publisher.
